# A Resilient, Non-neuronal Source of the Spatiotemporal Lag Structure Detected by BOLD Signal-Based Blood Flow Tracking

**DOI:** 10.3389/fnins.2017.00256

**Published:** 2017-05-11

**Authors:** Toshihiko Aso, Guanhua Jiang, Shin-ichi Urayama, Hidenao Fukuyama

**Affiliations:** Human Brain Research Center, Kyoto University Graduate School of MedicineKyoto, Japan

**Keywords:** BOLD contrast, cerebral blood flow, neurovascular coupling, resting-state fMRI, functional connectivity, image denoising, image reproducibility

## Abstract

Recent evidence has suggested that blood oxygenation level-dependent (BOLD) signals convey information about brain circulation via low frequency oscillation of systemic origin (sLFO) that travels through the vascular structure (“lag mapping”). Prompted by its promising application in both physiology and pathology, we examined this signal component using multiple approaches. A total of 30 healthy volunteers were recruited to perform two reproducibility experiments at 3 Tesla using multiband echo planar imaging. The first experiment investigated the effect of denoising and the second was designed to study the effect of subject behavior on lag mapping. The lag map's intersession test-retest reproducibility and image contrast were both diminished by removal of either the neuronal or the non-neuronal (e.g., cardiac, respiratory) components by independent component analysis-based denoising, suggesting that the neurovascular coupling also comprises a part of the BOLD lag structure. The lag maps were, at the same time, robust against local perfusion increases due to visuomotor task and global changes in perfusion induced by breath-holding at the same level as the intrasession reliability. The lag structure was preserved after time-locked averaging to the visuomotor task and breath-holding events, while any preceding signal changes were canceled out for the visuomotor task, consistent with the passive effect of neurovascular coupling in the venous side of the vasculature. These findings support the current assumption that lag mapping primarily reflects vascular structure despite the presence of sLFO perturbation of neuronal or non-neuronal origin and, thus, emphasize the vascular origin of the lag map, encouraging application of BOLD-based blood flow tracking.

## Introduction

Current functional magnetic resonance imaging (fMRI) technique, based on fluctuations in blood oxygenation level-dependent (BOLD) signals, are subject to various artifacts. While much is known about head motion, there are sources of confound related to blood flow and oxygenation dynamics: respiration (Birn et al., 2009; Birn, 2012), pseudo-positives from draining blood (Lai et al., 1993; Boubela et al., 2015) or mislocalization due to non-T2^*^ components (Wu et al., 2012; Kundu et al., 2014). Despite the inherent susceptibility of resting-state fMRI to both random and non-random noises, these confounds are easily or often overlooked because their impact is not always observable. It is therefore imperative to elucidate BOLD signal components that are uncoupled from neural activity to develop reliable biomarkers based on functional connectivity (FC).

Recently, a growing body of evidence has emerged supporting a spatiotemporally structured BOLD signal component that appears to reflect vascular anatomy. Since the introduction of fMRI, the impact of intravascular BOLD signals, which may lead to mislocalization, has been a major concern (Lai et al., 1993). It was subsequently found that the parenchymal fMRI signal is also not free from the vascular anatomy, causing variable latency (Chang et al., 2008; Wu and Marinazzo, 2016). This temporal lag structure can be imaged by mapping the phase of the systemic low-frequency oscillation (sLFO), which is also observable from non-brain body parts as a fluctuation of fMRI or a near-infrared spectroscopy signal (Anderson et al., 2011; Tong and Frederick, 2012). While the physiological mechanism underlying sLFO is yet to be fully understood, there are possible sources of temporal variation, such as blood oxygenation level, heart rate and blood pressure, that can concomitantly affect these signals (Tong and Frederick, 2014b). In parallel with the works on its physiology, a growing number of studies has sought a clinical application, taking advantage of the noninvasive nature of this blood tracking technique (Amemiya et al., 2013; Lv et al., 2013; Ni et al., 2017). The method, known as “lag mapping,” had only been validated in patients with cerebrovascular disease until recently, but has been confirmed in healthy controls to reflect the cerebral blood flow (Tong et al., 2016b).

According to the recent advances, the temporal lag structure accounts for ~30% of the signal variance on average, suggesting a profound impact of this lag structure on fMRI data (Erdoğan et al., 2016). In fact, it has been reported that effective connectivity analysis can be heavily contaminated by vascular structures when applied without caution (Taylor Webb et al., 2013). However, to date, although this time-lag structure is considered to be noise in fMRI (Chang et al., 2008; Tong and Frederick, 2010; Anderson et al., 2011), the extent to which it selectively reflects vascular structure is unknown. Theoretically, BOLD lag mapping should be sensitive to both perturbation of the sLFO time series in the brain, and its propagation pattern. For example, one cannot rule out the effect of the neuronal component within the global signal fluctuation (Schölvinck et al., 2010), which is often used as the reference time series to track sLFO. Moreover, many physiological parameters including heart rate and respiration, that should affect both sLFO and cerebral circulation, are also dependent on subject behavior (Birn et al., 2009). It has certainly been of fundamental importance in fMRI whether one can completely isolate neurovascular coupling from the BOLD signal: a thorough investigation of this spatiotemporal lag structure would be of significant interest.

In this study, a portion of which was presented previously as a poster (Aso et al., 2015), we aimed to investigate basic properties of this major confound in BOLD signal by testing the effect of various manipulations. The effect of fMRI denoising on the lag map was first assessed to confirm its non-neuronal sources. An independent component analysis (ICA)-based artifact removal with a set of simple heuristics, as one of the common approaches to fMRI denoising, was chosen. Test-retest reproducibility and image contrast were the quality measures examined. To minimize the number of potential sources of instability, we chose to introduce a temporally sparse task instead of performing the acquisition as complete resting state. The participants were given a low-demand, simple reaction time task (SRT) primarily to minimize variation from neural activity, including at vigilance level. Using this treatment, the reproducibility was accounted for mainly by non-neuronal confounders. Under these settings, the differential effects of denoising on FC and lag maps derived from the same dataset were investigated.

In contrast, for the second experiment, we manipulated the subject behavior to further assess the response of lag structure to BOLD signal perturbations. Respiration is one of the major sources of physiological noise, the manipulation of which is known to enable measurement of cerebrovascular reactivity according to BOLD signal (Kastrup et al., 1999; van Niftrik et al., 2016). If the lag maps simply reflect the vascular structure, the map would be robust to some degree against the modification of both sLFO and cerebral blood flow—either coupled or uncoupled to—neuronal activity. These two complementary approaches should help provide a better understanding of the mechanisms underlying the BOLD lag structure.

## Materials and methods

### Subjects and experimental procedures

Thirty healthy volunteers (six women; 20–29 years of age) participated in the study. Among them, 10 were specifically chosen from a list of volunteers who had recently participated in a study in our MRI scanner, given their excellent performance in terms of head stability, for the intersession reproducibility study. The other 20 volunteers participated in the breath-holding experiment. All of the subjects had no history of neurological or psychological problems, and written informed consent was obtained in a manner approved by the university medical school's institutional review board. To avoid vigilance level fluctuations, all of the MRI sessions were scheduled in the morning, and the participants were encouraged to sleep well the previous night.

### Image acquisition

A Tim-Trio 3 Tesla scanner (Siemens, Erlangen, Germany) with a 32-channel phased-array head coil was used to obtain all of the images. For the reproducibility study, T2^*^-weighted echo-planar images were acquired using multiband gradient-echo echo-planar imaging (EPI; Feinberg et al., 2010) with the following parameters to cover the entire cerebrum and dorsal part of the cerebellum: 64 × 64 pixels, interleaved 28 slices; 224-mm field of view (FOV); 4 mm slice thickness; repetition time (TR)/echo time (TE) = 500/35; and multiband factor of four. From each experimental session, two 5 min runs (624 volumes × 2) were subjected to analysis to enable both inter-session (day 1 vs. day 2) and intra-session (run 1 vs. run 2) comparisons. Only slightly different settings were used for the breath-holding experiment: 35 slices, 192-mm FOV, 3 mm slice thickness, multiband factor of five, and one 9 min run (1,080 volumes). After the main runs, a 3D T1-weighted image was acquired with the following parameters: TR = 1630 ms; TE = 4.38 ms; inversion time = 990 ms; FOV = 240 mm; voxel size = 0.94 × 0.94 × 0.95 mm; 8° Flip angle, and 130 Hz bandwidth.

#### Intersession reproducibility experiment

Volunteers underwent two MRI sessions separated by at least 6 days. During the two BOLD runs in each day, the subjects were given a sparse SRT with a varying inter-trial interval of 6–24 s. They were instructed to hold a button box while viewing a computer screen showing “Please hold still” and to respond as soon as the screen changed to “Press the button.” The screen returned to “Please hold still” at the button press or after 3 s if the participant did not press the button. The reaction time was recorded for performance evaluation.

#### Breath-holding experiment

The subjects underwent 4 runs each with different conditions: simple rest, a sparse SRT, animation viewing and brief breath-holding tasks. Animation viewing was not used for the present analysis. The screen showed “Please hold still” for the entire duration of the simple rest condition. The breath-holding task was cued by a “Hold your breath” instruction on the screen, at which point the subjects were asked to immediately hold their respiration irrespective of the respiration phase. The holding periods lasted for 10 s and were separated by 90 s intervals.

### Data preprocessing and ICA-based denoising

Images from each individual were treated as one dataset with 4 runs in the preprocessing and ICA-based denoising. The frames of the four-dimensional (4D) BOLD image data were first corrected for head motion using SPM (SPM8; Wellcome Department of Cognitive Neurology, London, United Kingdom) in MATLAB. After high-pass filtering at 0.008 Hz to remove slow drifts, the images were fed into the ICA pipeline without any spatial smoothing for efficient noise classification (Smith et al., 2012). MELODIC/FSL software, version 5.0 (FMRIB Software Library, http://www.fmrib.ox.ac.uk/fsl; Smith et al., 2004) was used to perform the individual ICA with automatic dimensionality estimation, identifying 102–144 components (mean 122.4).

Under the assumption that BOLD signal dynamics consist of spatially independent processes of various physiological and physical mechanisms, ICA has been used for cleaning fMRI data (Thomas et al., 2002; Perlbarg et al., 2007; Pruim et al., 2015). A simplified version of the noise classification strategy with a feature set was used, an approach used in previous studies (Thomas et al., 2002; Bhaganagarapu et al., 2013; Pruim et al., 2015). To quantitatively evaluate the likelihood of each component being noise, three measurements were obtained: high-frequency power fraction >0.2 Hz; non-gray matter involvement; and slice dependency. Non-gray matter involvement was calculated using the tissue segmentation results of each subject's anatomical T1 images (Ashburner and Friston, 2005). Note that the precision of tissue classification was limited by the noticeable partial volume effect with the low resolution of BOLD images. A weighted mask image was created as a summed image of tissue probability images of the gray matter (range 0–1) and white matter, divided by 2 to compensate for partial volume. Each independent component map was smoothed at an 8 mm full width at half maximum (FWHM) kernel, and was thresholded at z > 1 (or z < −1) before the mask was applied to calculate the mean gray-matter involvement index over voxels, which was then subtracted from 1 to yield the non-gray matter index. Slice dependency was defined as the ratio of the mean Fourier spectral power in space between the Z- and Y-axes to capture components with large variations between neighboring slices (e.g., with motion or mechanical origin).

**Figure 1:**
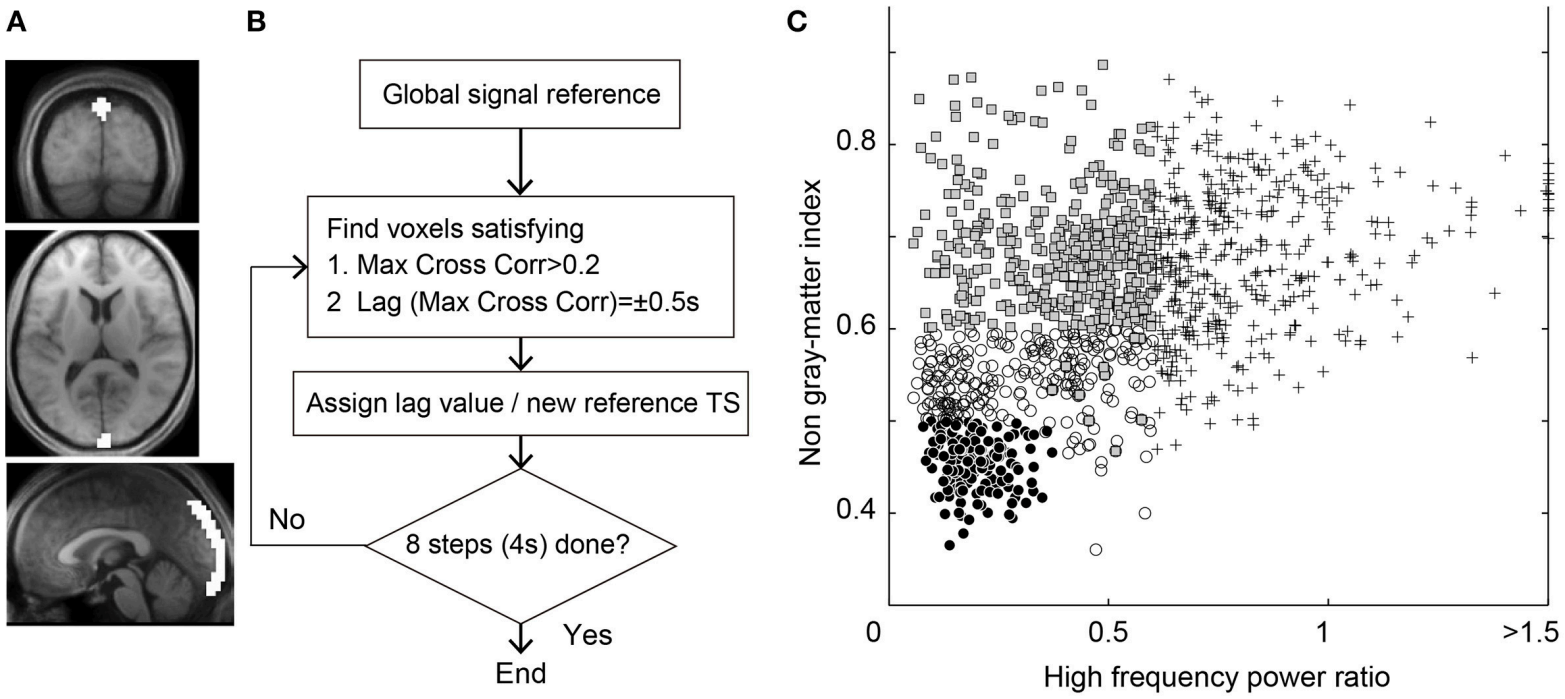
**(A)** Region of interest for the seed time series of Lag-SSS covering the posterior part of superior sagittal sinus. **(B)** Schematic diagram of Lag-rec mapping procedure. **(C)** Scatterplot of the 1,224 components from the individual ICA for denoising. Components regressed out in Dn1 owing to high temporal frequency power (crosses), those additionally removed in Dn2 (shaded rectangles) and in Dn3 (open circles), and the “signal” components surviving the Dn3 filtering (filled circles) are shown.

On obtaining the three parameters, components were classified as non-neuronal when at least one of the three measurements exceeded the respective threshold. The cutoff thresholds were determined on a purely arbitrary basis to achieve a stepwise increase in denoising strength (Figure 1C). The weakest denoising (Dn1) used only the high-frequency power index at a high/low-frequency power ratio >0.6, by which 34.8% of components on average were classified as noise. For moderate denoising (Dn2), 67.5% of components were removed by adding a non-gray matter involvement threshold of 0.6 and a slice dependency of four. Aggressive denoising (Dn3) removed 86.3% of components with a high-frequency power index of 0.4, a mean gray matter index of 0.5, and a slice dependency of 3. A noise-only dataset was also created by regressing out the non-noise components from the Dn3 procedure (Noise dataset). The regression in this denoising process involved all of the components, instead of aggressively removing the full space of the noise components (Griffanti et al., 2014; Pruim et al., 2015). An additional analysis with even lower denoising strength was performed using only the slice dependency to classify 15.1% of components as noise for confirmation of the main analysis.

After denoising, spatial normalization was applied to the Montreal Neurological Institute template, using the parameters obtained via the unified segmentation process described above. The first 20 volumes (10 s) were discarded from each run to exclude transition responses related to factors such as scanner noise. The time series of each voxel was then normalized to zero mean and unit variance before concatenating the two runs from each day (for inter-session comparison) or from the first and second runs of each day (intra-session). To focus on the effect of denoising, the remainder of the procedures were made uniform across mapping methods and datasets, including the band-pass filtering (0.008–0.16 Hz). For example, low-pass filtering may be redundant after sophisticated denoising (Smith et al., 2012); however, a conservative approach was chosen because our analysis included the raw dataset. Nevertheless, a wider band pass was used to track the lag, following earlier studies (Braun et al., 2012; Tong and Frederick, 2014b).

In the breath-holding experiment, which involved 20 subjects, both neural activity and physiological confounds were manipulated, no denoising was performed, and only lag maps were created (see below).

### Creating BOLD signal-based maps

#### Seed-based correlation mapping

The default mode network (DMN) and executive control network (ECN) maps were created using seed-based correlation mapping (Fox et al., 2005). The human ECN was originally defined by Seeley et al. (2007) as a system dissociable from the saliency network by the inclusion of a dorsal medial prefrontal cortex region, instead of the anterior cingulate cortex. These two networks were chosen because of their rich remote connectivity, high reproducibility in the general population (Smith et al., 2012), and mutually exclusive distribution. The seed region for the DMN was the posterior cingulate cortex, as defined in the automated anatomical labeling atlas (Tzourio-Mazoyer et al., 2002). For the ECN, a region of interest (ROI) was defined along the paracingulate sulci [centered on (±10, 24, 42)], based on the earlier studies. For the principal analysis, the data were not “cleaned” by regressing out white matter and cerebrospinal fluid (CSF) signals, as proposed in the classic FC studies. The effects of this common procedure have not been well-established in combination with ICA-based denoising techniques, which themselves can reduce non-gray matter components (Chang and Glover, 2009). Moreover, the present study explicitly assumed that those signals contain information about blood circulation; therefore, the cleaning procedure could directly affect lag mapping. Consequently, white matter/CSF signal regression was tested solely for confirmation of the denoising effect on the FC maps.

#### Lag mapping

Two types of lag mapping techniques were used: Lag-SSS, a simple seed-based lag mapping; and Lag-rec, a recursive tracking method. For Lag-SSS, an ROI along the descending part of the superior sagittal sinus (SSS) was used to obtain the reference time course with which the time lags in all of the voxels were measured (Figure 1A; Christen et al., 2015). The lag maps were created by calculating the time shift relative to the SSS signal, which yielded the maximum of the correlation coefficients for each brain voxel. This procedure was actually performed by creating a 3D array with dimensions of time × space (voxel) × time-shift and by calculating the maxima along the third dimension. Positive values were assigned to the upstream (i.e., earlier arrival of the labeling component of the signal) voxels providing a majority of the brain regions' positive values. The lag map took discrete values between −4 s and +6 s at the interval of TR (= 0.5 s), which was then spatially smoothed at 8 mm FWHM.

In contrast, in Lag-rec, the reference signal was updated recursively (Tong and Frederick, 2014b). A schematic diagram of the procedure is shown in Figure 1B. In each step, a cross-correlogram was calculated with the seed signal to obtain a set of voxels with a local peak at a time lag of ±0.5 s, which then served as the new seed region. The global mean signal was used to select the initial seed, indicating that the voxels presenting maximal correlation with the global signal at zero time shift served as the first seed. In this recursive method, the tracking part left some voxels without any lag values because the cross-correlogram peak <0.2 was discarded (Tong et al., 2016b). These “holes” in the resulting lag maps were single isolated voxels in most cases and filled using a partial differential equation-based interpolation (Huang et al., 2005). The lag was tracked up to 4 s in both directions: up- and down-stream.

### Effect of TR on reproducibility

To investigate the differential effect of sampling interval on the BOLD-based metrics, each 4D dataset was decimated at every 2–10 time points to achieve varying effective TRs from 1 to 5 s. Because the MR signal time course cannot be filtered before sampling, unlike electroencephalogram, the BOLD signal is inherently subject to contamination from physiological noise from an aliasing effect. To observe the confounding effect of aliasing on the BOLD image analyses, the situation was intentionally simulated by applying no high-pass filtering before the decimation (Yang et al., 2007).

### Statistical analysis

Intra-class correlation coefficient (ICC; Raemaekers et al., 2007; Thomason et al., 2011) was used to evaluate test-retest reliability as consistency of measurement. Two measures of reproducibility were computed: one for stability of the images and one for repeatability as a measurement (Raemaekers et al., 2007). To facilitate comparison, the seed regions in the DMN and ECN were masked out beforehand. According to McGraw and Wong (1996), an ICC via two-way, mixed, single-measurement testing for absolute agreement of two measurements was formulated as

$$\begin{array}{l} ICC\left(2,1\right)=\frac{MS_{R}-MS_{E}}{MS_{R}+MS_{E}-2\left(MS_{C}-MS_{E}\right)/n} \end{array}$$

where *MS*_*R*_*, MS*_*C*_, and *MS*_*E*_ represent mean squares for rows, columns, and errors obtained from repeated-measures ANOVA, respectively. The number of columns is denoted as *n*. In the case of image reproducibility between the two sessions, image data were arranged in a matrix with sessions (days) as rows and voxels as columns (ICC_between_; Raemaekers et al., 2007). Absolute agreement across measurements within each voxel was calculated to create reproducibility maps by treating the subjects as columns (ICC_within_; Thomason et al., 2011). The results were statistically compared using the nonparametric Friedman test. For the breath-holding experiment, ICC_within_ was calculated using three measurements (rest, SRT and breath holding).

## Results

### Stability of the sparse SRT task performance

The mean response times were 603 ± 169 and 631 ± 134 ms for the first and second days, respectively. The Friedman test failed to detect significant session effects (*P* = 0.21).

### BOLD-based maps and the effects of denoising

**Figure 2:**
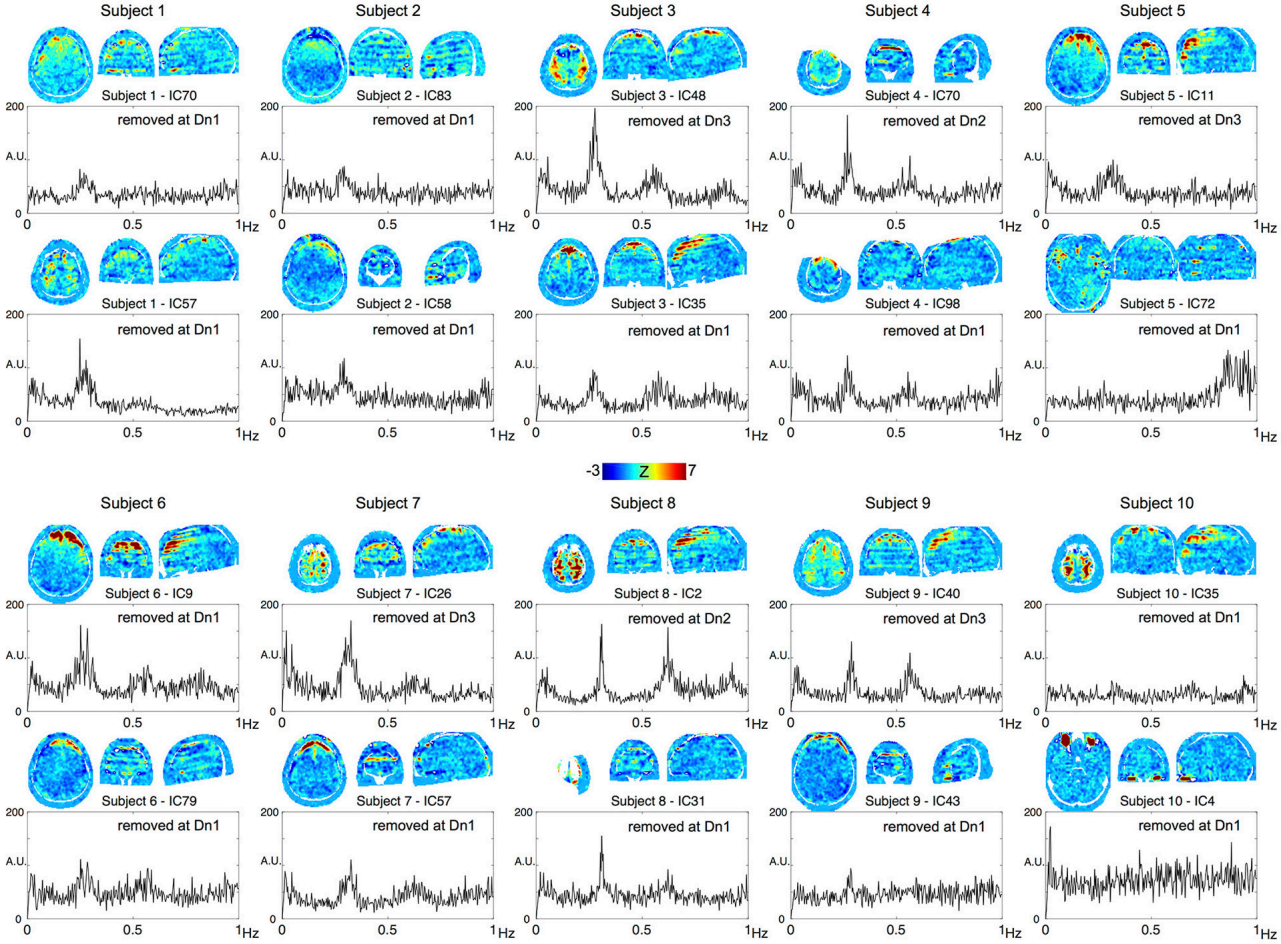
**The top two components from each subject, with the highest contribution to the spectral power between 0.2 and 0.3 Hz are shown**. All of these components were removed at different denoising levels as indicated in the panels.

To confirm the performance of the denoising algorithm, spatial distribution of the noise components removed in each of the three denoising strengths was first investigated (Supplementary Figure 1A). Throughout the three levels, the noise components tended to involve the brain surface, presumably owing to the motion-related artifacts or pial vein signal. Components removed at the most aggressive level (Dn3) did not involve the white matter or scalp, indicating that noise components involving these regions had already been captured at the lower levels. Among the additionally removed noise components, 4.8% exceeded the criteria for slice dependency at Dn2 and 10.9% at Dn3. Most of these slice-dependent components demonstrated the involvement of multiple (typically 28/4 = 7) slices uniformly separated from one other, possibly related to the simultaneous acquisition by multiband sequence (Feinberg et al., 2010). The effect of the denoising on global time series spectra is shown Supplementary Figure 1B. Reduction of high-frequency components >0.8 Hz is evident in Dn1, while the effect of denoising was most evident in the “hump” at ~0.2–0.3 Hz, noted previously (Smith et al., 2013; Tong and Frederick, 2014a). To trace their origins, components from the individual ICA were investigated. Shown in Figure 2 are the top two ICA components from each subject, with the highest ratio of the integral of spectral power between 0.2 and 0.3 Hz to that below 0.1 Hz. Many of these components contributing to the hump presented a unique spatial distribution with inter-individual variation in peak frequency, both of which are characteristic of respiration signals (Tong and Frederick, 2014a). The existence of harmonics and the alternating bands parallel to the acquisition slices would further suggest respiration-related, periodic motion as the main source of the signal. All of these ICA components were classified as noise, but at different denoising levels. Similarly, many of the components substantially contributing to the 0.8–0.9 Hz power coincided with the CSF voxels along the internal carotid and middle cerebral arteries, possibly representing cardiac pulsation (Supplementary Figure 2).

**Figure 3:**
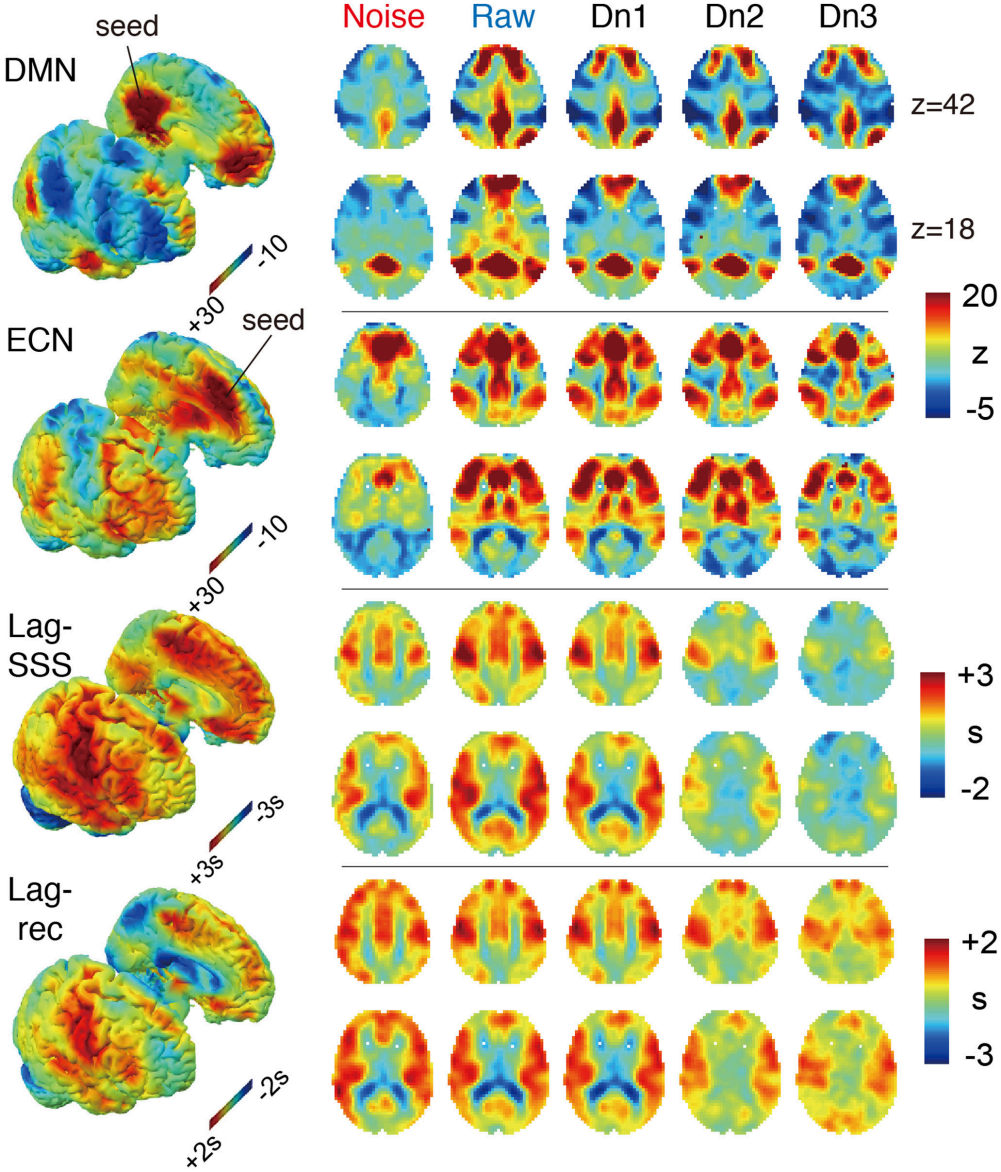
**The four BOLD-based metric maps investigated in the study and the effect of denoising intensity on the images**. In the 3D rendered brain on the left, the right and left hemispheres are split to show the medial surface. For the Lag maps, we assigned positive values to the voxels with earlier phase to make the map represent travel time to the global signal phase. Both FC maps presented higher specificity with more contrast between the members and non-members of the networks. Denoising had the opposite effect on the two lag maps, which was most prominent in the noise-only dataset (Noise), which showed similar results to the raw maps.

The effects of denoising on the group-averaged images are shown in Figure 3. The three FC maps present narrower and more defined clusters as more noise components were eliminated. Decreased correlations outside of the network hubs also resulted in extension of the negatively correlated areas, precisely consistent with earlier reports using different denoising techniques (Chang and Glover, 2009). On the two lag maps, there were opposite effects that dramatically reduced the contrast and thus the lag structure, suggesting the failure of blood tracking in “cleaned” data. It is important to note that lag maps in the current study were created to represent phase advance instead of delay, thus resulting in opposite polarity to earlier works. Hence, the voxel values indicate the relative “drainage time” in reference to the seed. This choice was motivated by the fact that lag structure is sensitive to the venous side of the circulation (Tong et al., 2016b). The borderzone sign, which reflects delayed arrival in the border between the arterial territories (Zaharchuk et al., 2009), is found as the area of small phase advance for gray matter.

### Effects of denoising on reproducibility

#### ICC_between_

**Figure 4:**
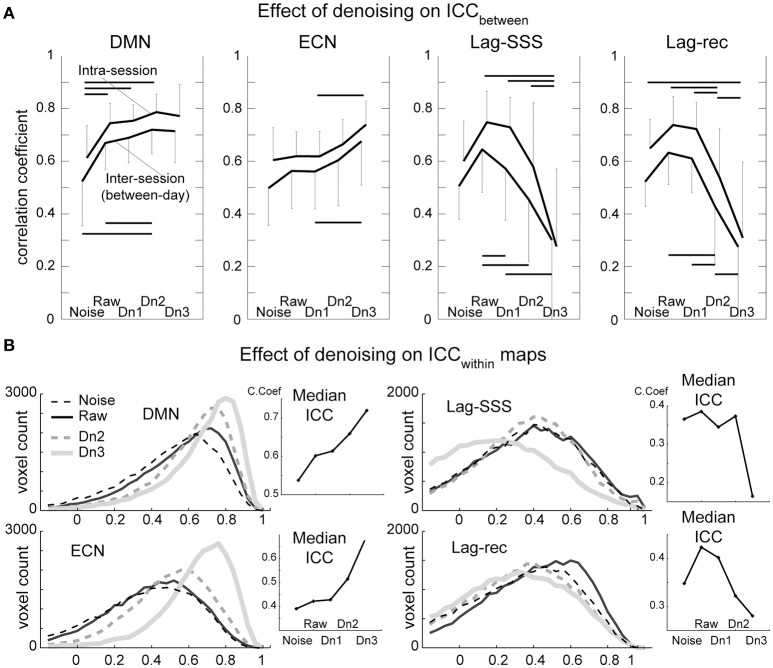
**Results from the reproducibility experiment**. **(A)** Test-retest reliability and denoising. Image similarity quantified by ICC_between_ is plotted against denoising strength. FC maps became more stable by increasing denoising strength, whereas lag maps presented the highest reproducibility in the raw dataset. The overall effect of denoising on reproducibility was significant for all four BOLD metrics (Friedman test, *P* < 0.001) except for the ECN (*P* = 0.07). Error bars signify 95% confidence intervals (*n* = 10). Horizontal bars denote a significant difference (*P* < 0.01) by the paired *t*-test. **(B)**. Reproducibility of measurements within each voxel (ICC_within_) is shown in histogram form. This analysis revealed superior performance of aggressive denoising (Dn3) over the moderate level (Dn2) on FC maps. For lag maps, the effect of denoising was the opposite.

Test–retest reliability has been characterized as excellent (ICC > 0.8), good (ICC 0.6–0.79), moderate (ICC 0.4–0.59), fair (ICC 0.2–0.39), or poor (ICC < 0.2; (Birn et al., 2014)). As shown in Figure 4A, all four of the BOLD-based metrics evaluated here showed moderate to good ICC_between_ values in inter-session comparisons, whereas intra-session ICC yielded good peak reproducibility after optimal denoising. This difference between the inter- and intra-session reproducibility was highly significant (*P* < 0.0001; Friedman's test), indicating between-day component of variation.

As expected, there was a tendency for FC maps to be more reproducible as more “noise” components were eliminated. However, the ECN map failed to present significant effects of denoising over the five levels, owing primarily to inter-individual variation in the effects of moderate denoising. Within a subset of the results (noise, raw, and Dn3) used, the effect attained statistical significance (*P* = 0.02). The results were essentially similar with or without white matter/CSF signal removal (Supplementary Figure 3).

The lag maps, in contrast, presented moderate to good reliability before denoising and became completely unstable after aggressive denoising. This finding was observed in parallel with the loss of lag information from denoising in Figure 3. The lag maps' reproducibility was greatest with the raw dataset because, interestingly, removal of the non-noise components also diminished the ICC_between_. After failing to find any improvement by denoising, the additional analysis was conducted using the weakest denoising strength, based only on slice dependency to capture motion-related or mechanical noises. It again resulted in significant degradation of reproducibility in Lag-SSS (*P* < 0.01) and no improvement either in Lag-rec.

#### ICC_within_

Although the overall effect of denoising on within-voxel reliability was similar to that of ICC_between_, it was significantly more sensitive to cleanup by the most intensive denoising, Dn3, as depicted in the plot of median ICCs across voxels (Figure 4B). This effect was also remarkable in terms of the loss of reliability in the lag maps. The raw dataset was the most reliable for the lag maps, in agreement with the findings for ICC_between_.

### Lag structure and its stability against blood flow perturbation

#### Lag structure

**Figure 5:**
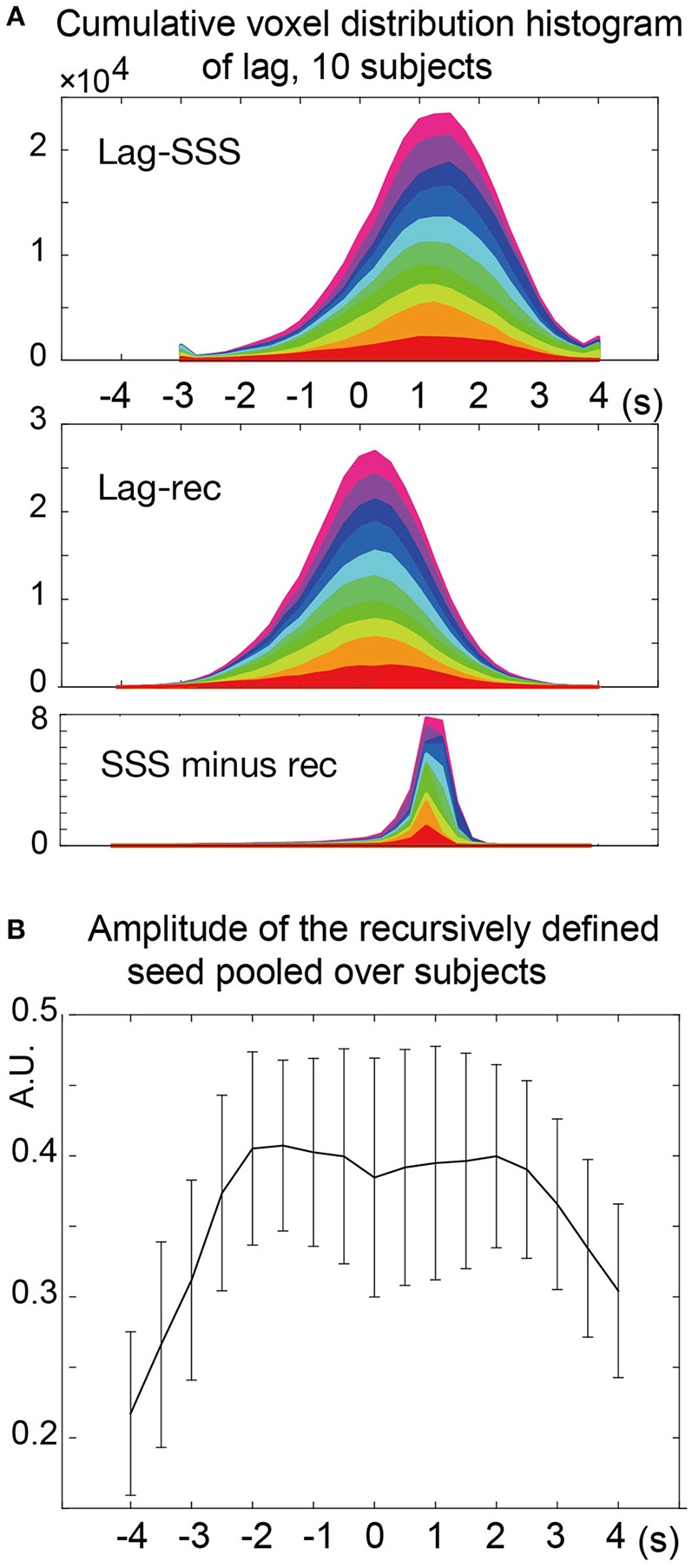
**Analysis of the lag maps. (A)** Voxel frequency histogram of the two lag maps and their difference map. The narrow shape of the difference map histogram suggests a common lag structure detected by the two algorithms. Colors represent the 10 subjects. **(B)** Amplitude (standard deviation) of the recursively defined reference time courses. There was a reduction in the lag-encoding component magnitude beyond 2 s in both up- and down-stream directions, suggesting loss of information.

The two types of lag maps showed different lag values but with similar structures (voxel-by-voxel, Pearson's correlation coefficient = 0.83 ± 0.08, 20 pairs of lag maps, mean ± *SD*). This finding was confirmed by creating a voxel histogram (Figure 5A). Most of the brain voxels in the Lag-SSS maps showed positive values consistent with their positions upstream of the SSS. When the Lag-rec maps were subtracted from the Lag-SSS maps in every subject, the histogram from the resulting images had a narrow shape, peaking at ~1 s.

**Figure 6:**
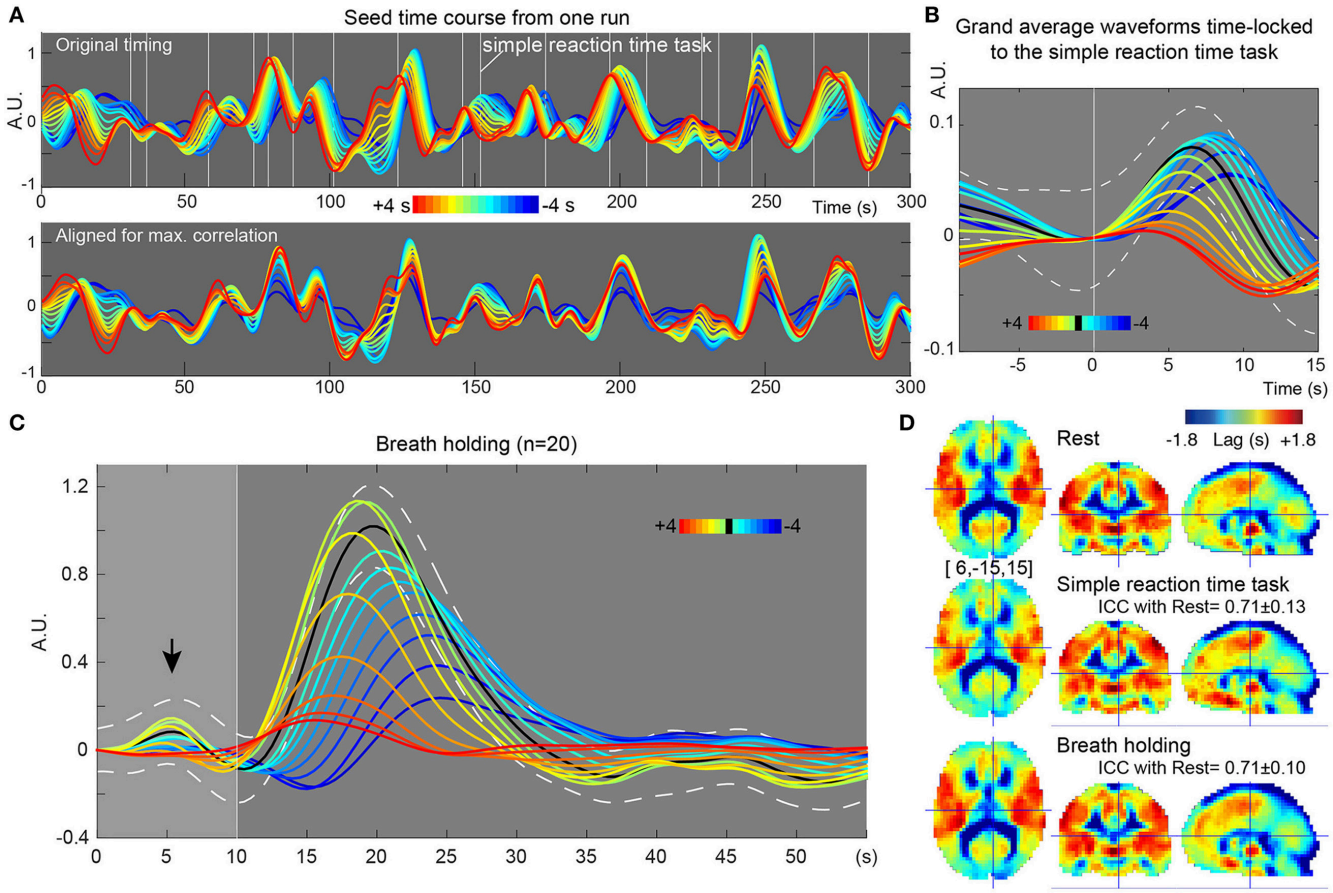
**Results from further evaluation of lag maps (breath-holding experiment)**. The color maps were chosen to represent more upstream (i.e., arterial) structure by warm colors and venous side by cool ones. The y-axes of the plots display relative MR signal intensity in arbitrary unit, which should be proportional to the normalized percent signal change. **(A)** Recursively generated seed-time courses on Lag-rec mapping from a single session of one subject. The phase is first tracked back from the global signal (green sweep) to 4 s earlier (red) and then tracked forward to −4 s (blue), thus expressed in the opposite polarity to the convention used in some previous works. Note that blood flow-related information is emphasized in these time courses by pooling signals from voxels sharing lag phase. White vertical lines indicate the stimulus presentation of the infrequent SRT task. **(B)** The time courses were subjected to time-locked averaging at the SRT task event and then grand averaged over participants. Only events separated from the previous trial by 9 s and from the following trial by 15 s were used. The black line indicates the first seed signal of lag = 0, which is almost identical to the global signal change time series (white broken lines denote a 95% confidence interval of the mean across subjects). The flow-related signal components were dispersed by this time-locked averaging in the upstream voxels, indicating that the neuro-vascular coupling is modifying the lag structure mainly in the venous side. **(C)** Seed time courses time-locked to the brief breath holding, pooled over the 20 participants. **(D)** Average Lag-rec maps for the three conditions are presented. ICC_between_ indicated good reliability of the Lag-rec map in spite of the perturbation by tasks. ICC_between_ between SRT and breath-holding was 0.75 ± 0.08.

In the recursive method of lag mapping, the reference signal updated at each step of lag tracking could be extracted. First, the seed signal amplitude in each part of the lag map was measured, in percent signal change normalized to global mean (Figure 5B). The magnitude of the seed signal was smaller in the upstream voxels (*P* < 0.0001; one-way ANOVA), and it peaked at the downstream seed. Note that the signal amplitude was already diminished at 4 s, suggesting that lag tracking was less reliable beyond this range. As depicted in Figure 6, the labeling component became visible by overlaying the seed-time courses. The arrival timing of the component over brain regions are reflected as phase variations of the curves in the Figure 6A. To verify the effects of neurovascular coupling on lag tracking, the time-locked response of each of these curves to the SRT was calculated (Figure 6B). There was a global response (black line) to infrequent SRT events, which although small (~0.06%), was still visible by grand averaging. The recursively defined seed signals in the upstream direction were quickly dispersed or averaged out by this procedure (reddish sweeps). We further confirmed that denoising eliminated this lag structure, along with the global response itself (Supplementary Figure 4).

#### Stability of lag map across different conditions

**Figure 7:**
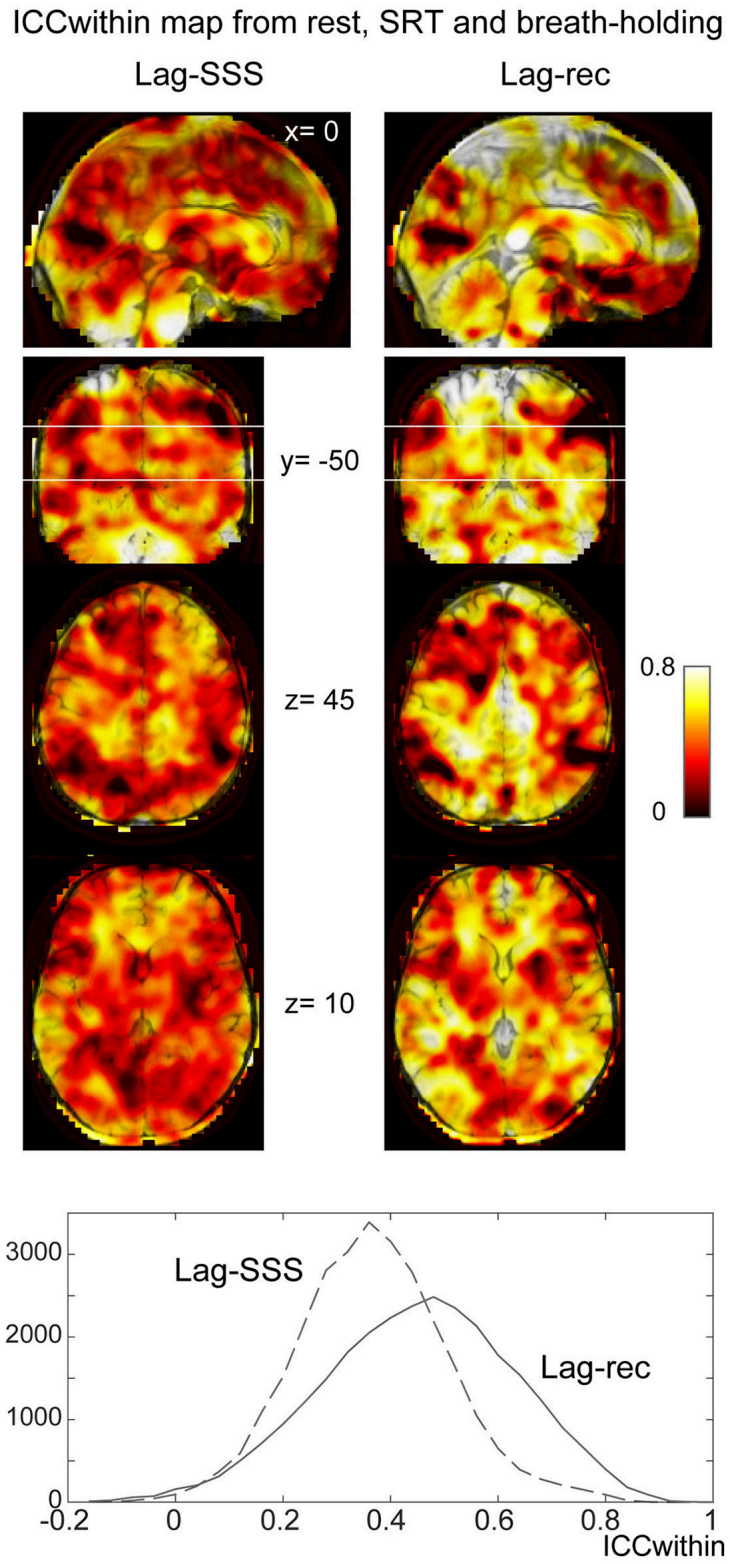
**Within-voxel reproducibility from the breath holding experiment**. ICC_within_ was calculated using the three runs: rest, SRT task, and breath-holding. It is clear from the maps and the image histogram that reliability of the Lag-rec map was superior to the Lag-SSS, although they shared some spots of poor reproducibility.

As shown in Figure 6C, the 10 s brief breath hold caused a delayed global signal increase via vasodilation (Kastrup et al., 1999) with higher amplitude, compared with the SRT task response (Figure 5B). After breath-hold onset (arrow), the global signal exhibited a small peak at 5 s, presumably due to neurovascular coupling (see Figure 6B). The lag maps were preserved despite the perturbation from task or controlled respiration with good reproducibility in ICC_between_ (Figure 6D). ICC_within_ from this experiment confirmed the results of the reproducibility experiment, but with strikingly higher stability of Lag-rec measurement (Figure 7).

### Effect of sampling rate on reproducibility

The effect of the simulated sampling rate from decimation was evaluated using inter-session ICC_within_ and image preservation, or similarity with the original dataset at TR = 0.5 s (Supplementary Figures 5, 6). Both the ICC and image preservation showed effects of TR (*P* < 0.01 for image preservation; Friedman's test), which were pronounced in lag relative to the FC maps (all combinations between either DMN or ECN vs. either Lag-SSS or Lag-rec; *P* < 0.001). Interestingly, there were “notches” at 1 and 2 s in both measures for the lag maps, creating a jagged pattern with long error bars indicating additional sources of signal instability at these sampling rates.

## Discussion

The major findings of the present study are as follows: time-lag structure within the BOLD signal is sensitive (or vulnerable) to ICA-based denoising designed to remove non-neuronal confound, losing both information and reproducibility; and lag maps created from the raw signal presented good inter-session reproducibility and were robust to subject behavior that causes local (i.e., SRT) or global (i.e., breath-holding) perfusion changes.

We first observed that the lag map lost both information and reproducibility by the denoising. This effect of denoising, which was essentially opposite to that of FC maps, is consistent with the non-neuronal origin of the lag structure. Another intriguing finding was that the lag structure was most stable in the “raw” signal without any ICA decomposition. Because the removal of “neuronal” components reduced the reproducibility, either neurovascular (or neuro-BOLD) coupling may also comprise a part of the lag structure. This is compatible with the largely passive nature of the neuro-BOLD coupling processes (Malonek et al., 1997; Chang et al., 2008) and the broad impact of lag structure in the fMRI signal as reported (Erdoğan et al., 2016). We also found that the structure is robust to the detection algorithms, again supporting the resilient nature of this component.

It was particularly interesting to observe that ICA-based denoising always resulted in diminished reproducibility compared with the Raw dataset, even at the weakest level. It makes a good contrast with the favorable effects on FC maps. Simply regressing out the time series from the respiration or cardiac components may have impaired the lag structure as there is considerable amount of low-frequency power that can be attributed to the sLFO (Tong et al., 2013). However, one should note that there may be a limitation of ICA itself because the underlying assumptions may not hold in some cases (Rajapakse et al., 2002). In fact, at the same time, we observed a clear effect of TR or sampling interval on the quality of lag mapping, which suggests room for improvement in lag map's reproducibility. This effect is possibly related to aliasing of cardiac rhythm because the heart rate in young adult positioned supine is often close to 60 beats/min (Budgell and Hirano, 2001; Watanabe et al., 2007), or 1 Hz, leading to higher probability of low-frequency contamination at the TR of 1 s (Cordes et al., 2014; Liang et al., 2015). Although further investigation is warranted, selective removal of the aliased cardiac rate using simultaneous physiological measurement could therefore improve the stability of lag mapping.

We also found that the reproducibility of the lag maps created from the Raw dataset was comparable with that of the FC maps created from cleaned dataset. The direction of blood flow is believed to be reversible across collaterals because the cerebral veins have no valves, and very acute changes in the outflow pathway have been reported (Gisolf et al., 2004). Hence, vascular topology in the sense of flow pattern can change. From the marked improvement in reliability from combining runs from different days (i.e., intrasession > intersession ICC), there may be both short- and long-term fluctuations in cerebral blood flow, as observed in blood pressure (Schillaci et al., 2012).

The second experiment used ICC as a direct measure of stability, instead of a quality measure. It demonstrated robustness of lag maps against the influence of manipulations in subject behavior, which further suggests that the lag structure, detected as a propagation pattern of the sLFO waveform, primarily reflects vascular anatomy. This low-frequency fluctuation that travels through the brain is believed to be largely of systemic origin and intrinsic to blood (Tong et al., 2016b) such as oxygen content (Rostrup et al., 1995) or cardiac output (Erdoğan et al., 2016). The robustness of lag maps has already been postulated by the reliability of this technique in detecting pathological perfusion delay with very small segments of BOLD time series (Amemiya et al., 2013), but not been confirmed to hold true also in the physiological range. The time-locked response of the seed time courses to the SRTs revealed how neurovascular coupling is embedded in the lag structure. As predicted by Tong and Frederick (2014b), there was higher reproducibility of Lag-rec over Lag-SSS, suggesting a fluctuation of propagating sLFO which is compensated for by the adaptive approach. One possible source of this fluctuation may be neurovascular coupling as observed in both SRT and breath-holding conditions, with small but widespread aftereffect (Boubela et al., 2015); however, further studies are needed for a fuller picture.

Findings of the present study have additional implications for common data-cleaning procedures using linear regression. The lag structure maps revealed a clear anatomical and, thus, tissue correspondence of blood circulation, as reported previously (Taylor Webb et al., 2013; Tong et al., 2016a), meaning that the white matter or CSF time course could contain traces of cortical activity from a range of brain regions, depending on the venous drainage route. Simply regressing out these BOLD time courses would create spurious correlation enhancement or reduction depending on the relative phase of each voxel (Erdoğan et al., 2016). An interesting future direction would be to incorporate the lag structure detected by a recursive procedure to model the signal response for fMRI analysis.

To the best of our knowledge, this study is also the first to demonstrate improvement in the test-retest reliability of FC maps via ICA-based denoising. In fact, test-retest reliability has not been evaluated in relation to the preprocessing technique until recently (Birn et al., 2014; Shirer et al., 2015). Reproducibility is a prerequisite for a useful biomarker—as it is for any scientific test—and it is critical for an “orphan” biomarker such as BOLD-based connectivity, that does not allow for straightforward validation using other techniques. Of course, high reliability alone does not necessarily indicate successful measurement (Braun et al., 2012). In the present results, for example, the ECN map failed to present significant drops in reliability in the noise dataset, despite considerable degradation of the map itself. Conversely, the greatest reliability in the Dn3 dataset may have been achieved at the expense of removing useful information. Using a similar denoising technique, Pruim et al. (2015) manually identified approximately three-quarters of the ICs as noise, midway between our Dn2 and Dn3. Needless to say, achieving maximum reproducibility while detecting the target biological parameters is desirable. Currently, because there is no gold standard for FC map quality, identification of non-neuronal components would serve as a helpful strategy. In fact, information extracted by the lag mapping is relatively straightforward because it is quantitative (in seconds) and enables validation using other techniques (Christen et al., 2015; Tong et al., 2016b). Elimination of both information and reliability of the lag maps by denoising in the present study at least strongly encouraged sufficient removal of these components to achieve high level of neuro-BOLD coupling (Erdoğan et al., 2016). In relation, higher spatial resolution will improve the characterization of noise sources because the lag structure should be spatially coarser and, as we experienced in the present study, tissue classification for denoising is affected by partial volume in the original BOLD image. The overall findings suggest that optimized spatial and temporal resolutions should be combined with a denoising algorithm to achieve higher level of neuro-BOLD coupling.

## Conclusion

Results of the present study add to the recently growing recognition of the propagating low-frequency fMRI signal component of systemic origin. Consistent with earlier reports, our findings further emphasize the purely non-neuronal origin of this lag structure by degradation of both the map's information and reproducibility in parallel with the strength of noise removal, despite the use of independent denoising approach from the lag mapping procedure. This time lag map should primarily reflect the vascular anatomy, judging from its stability against local (vasomotor task) or global (breath-hold challenge) perfusion changes, while it should also be sensitive to change in CBF dynamics such as instantaneous variation of transit time from artery to vein, for example. There was also a gradual change over vascular structure in the waveform of the low-frequency component, resulting in superior reproducibility of the map created by an adaptive, recursive procedure. Neurovascular coupling should be contributing to this fluctuation of waveform; however, further investigation with higher spatial resolution is needed to fully address the relationship between these two, in earnest with the ultimate goals of complete removal of non-neuronal BOLD components in fMRI and reliable blood flow tracking by lag mapping.

## Ethics statement

Ethics Committee of Kyoto University School of Medicine. The volunteers provided their written informed consent for the analysis of anonymized MRI scans and associated clinical data prior to the scan and all methods were carried out in accordance to the approved guidelines.

## Author contributions

All of the authors approve the manuscript version to be published. TA contributed to the conception and design of this research, data analysis and interpretation, and drafting of the manuscript. GJ contributed to data acquisition and manuscript draft. HF and SU contributed to the conception and design of this research, as well as data acquisition and interpretation.

## Funding

This work was supported by a grant from the Japan Society for the Promotion of Science (Grant-in-Aid for Scientific Research on Innovative Areas, 4703 and Grant-in-Aid for Scientific Research C, 25461817), Smoking Research Foundation and Takeda Science Foundation.

### Conflict of interest statement

The authors declare that the research was conducted in the absence of any commercial or financial relationships that could be construed as a potential conflict of interest.
