# Supplementary material for: A Resilient, Non-neuronal Source of the Spatiotemporal Lag Structure Detected by BOLD Signal-Based Blood Flow Tracking
Source: Front Neurosci. 2017 May 11;11:256. doi: 10.3389/fnins.2017.00256 (PMC5425609; doi:10.3389/fnins.2017.00256)
Supplement: Supplementary file 1 [file DataSheet1.pdf]

Supplementary Material

A resilient, non-neuronal source of the spatiotemporal lag structure detected by BOLD signal-based blood flow tracking

Toshihiko Aso, MD, PhD, Shin-ichi Urayama, MS, Hidenao Fukuyama, MD, PhD

Human Brain Research Center, Kyoto University Graduate School of Medicine, Kyoto, Japan

* **Correspondence:** Toshihiko Aso, aso.toshihiko@gmail.com

1.1 Supplementary Figures

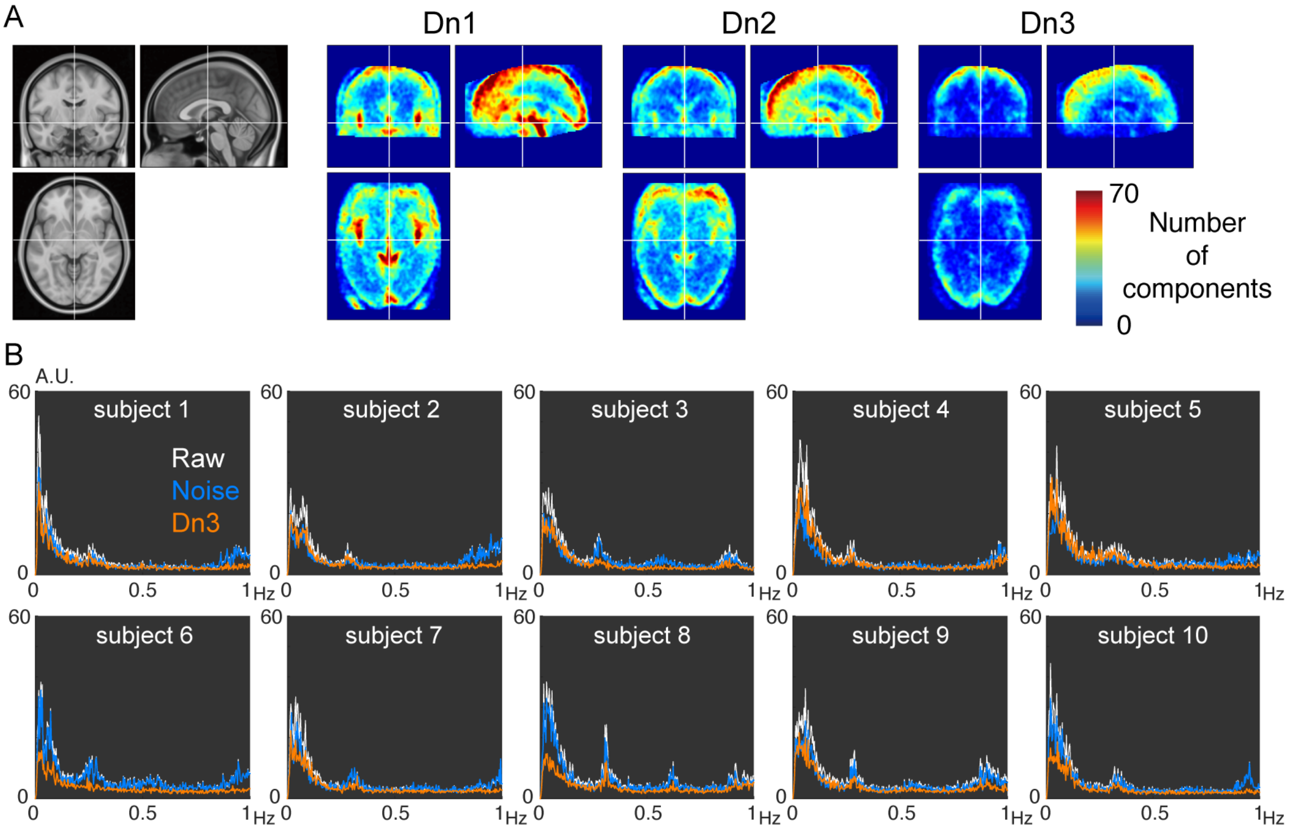

Supplementary Figure 1. A, ICA components classified as noise. From each denoising level, additionally removed components were summed after binarizing at a threshold of z = 2 to visualize the number of components involving each voxel. Because the denoising was performed in the original spaces of individual subjects, the components were spatially normalized solely to create these maps. B, Effect of denoising on spectra of the global signal timeseries. The spectra are averaged across all runs for each subject of the reproducibility experiment.

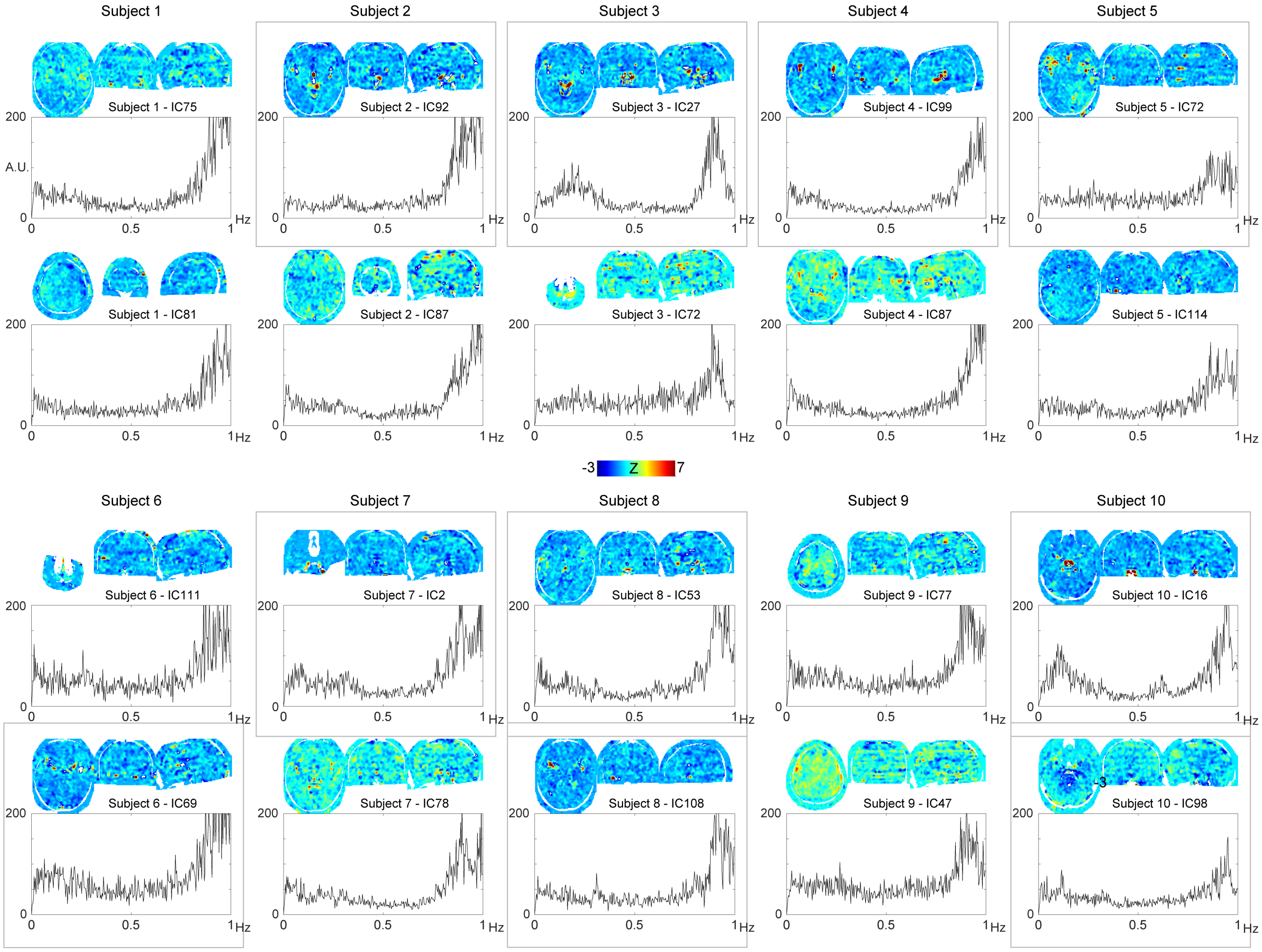

Supplementary Figure 2. From the individual ICA for denoising, the top two components with the highest ratio of the integral of spectral power between 0.8 Hz and 0.9 Hz to the integral of power below 0.1 Hz are shown. Components with characteristic spatial patterns are framed to indicate cardiac pulsation-related artifact. All of these components were classified as noise at the weakest denoising level (Dn1).

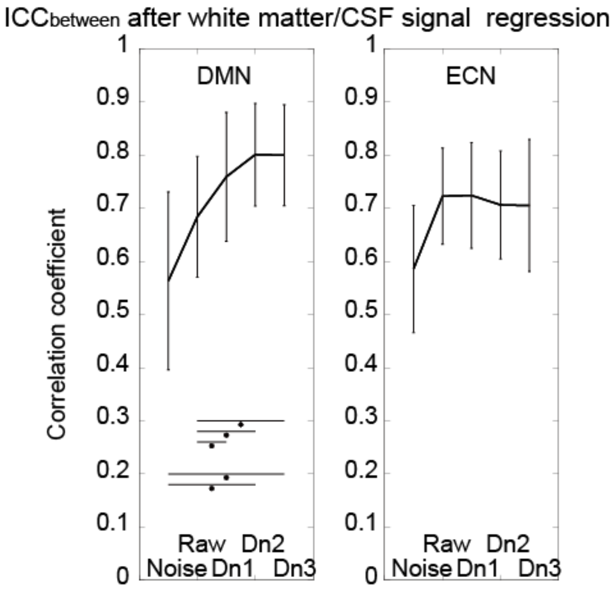

Supplementary Figure 3. Additional analysis of the effect of denoising on FC-map reproducibility with white matter and CSF regression. The result was essentially similar to the main analysis without regressing out these confounds.

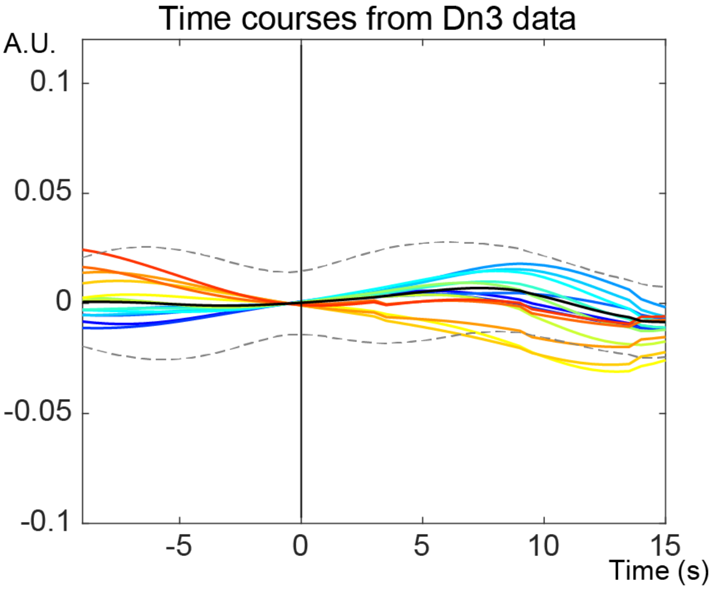

Supplementary Figure 4. Loss of lag structure after denoising. In the same convention as in Fig. 4B, response to the SRT task (at t=0) is presented. Since the lag map cannot be reliably produced from this dataset, Lag-rec map from the Raw dataset was used to select voxels to extract the time courses. Broken lines denote a 95% confidence interval of the mean global signal (black) across subjects.

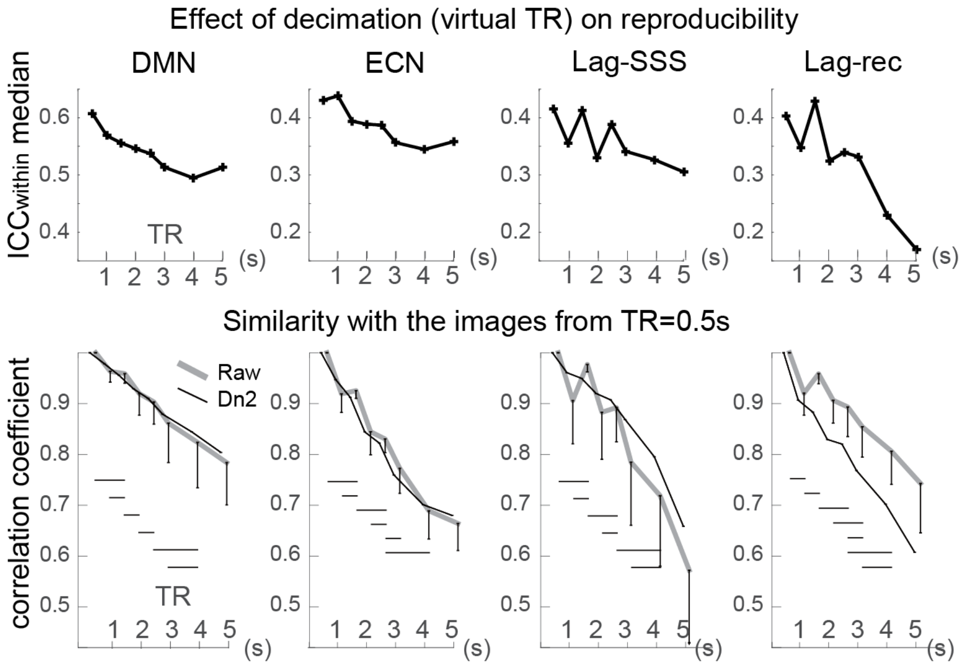

Supplementary Figure 5. Effect of sampling rate on ICC_{within} and image preservation. Decimation (resampling) was performed at every two, three, four, five, six, eight, and 10 time points to achieve virtual TR from 1 s to 5 s. For the two FC network maps, image preservation was evaluated using Pearson's correlation coefficient maps, instead of Z scores, because of varying degrees of freedom. Horizontal bars indicate significant differences by paired t testing at $P < 0.01$. At harmonic frequencies of 1 Hz, there was diminishment of both reproducibility and image preservation.

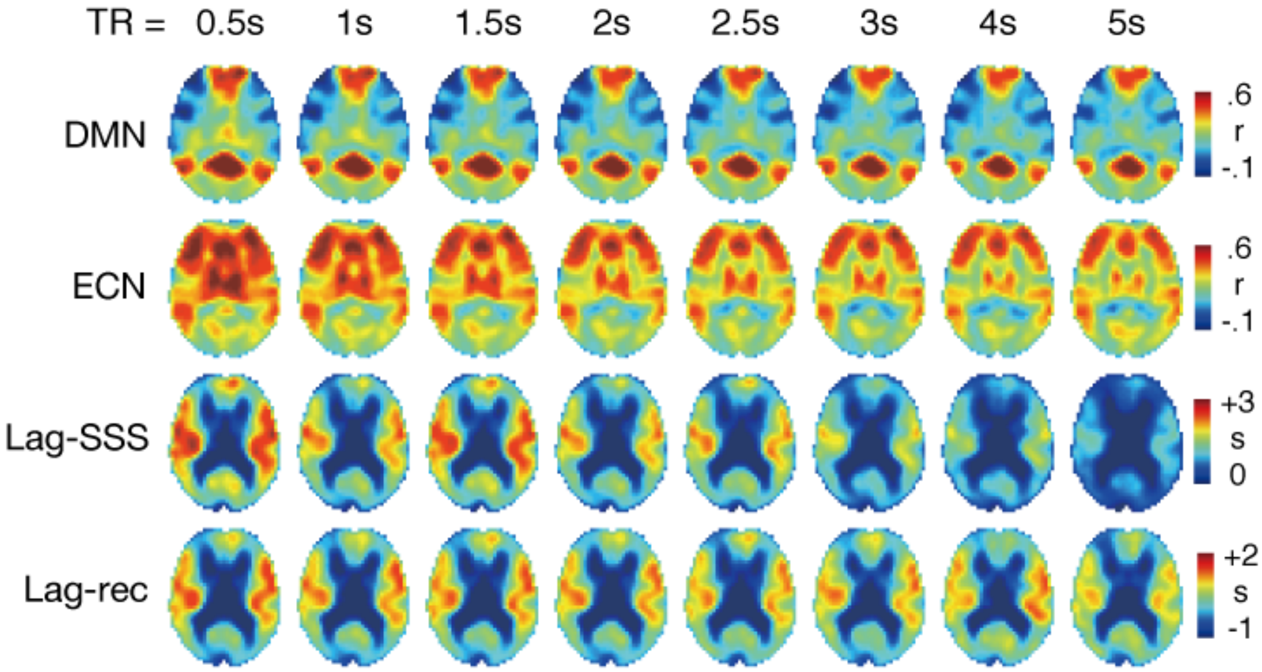

Supplementary Figure 6. The original images used to create the bottom panels of Supplementary Figure 5. FC maps are not converted into z because of different degrees of freedom.
